# Evaluating health service coverage in Ghana’s Volta Region using a modified Tanahashi model

**DOI:** 10.1080/16549716.2020.1732664

**Published:** 2020-03-16

**Authors:** Mallory C. Sheff, Ayaga A. Bawah, Patrick O. Asuming, Pearl Kyei, Mawuli Kushitor, James F. Phillips, S. Patrick Kachur

**Affiliations:** a Heilbrunn Department of Population and Family Health, Mailman School of Public Health, Columbia University, New York, NY, USA; b Regional Institute for Population Studies, University of Ghana, Accra, Ghana; c Department of Finance, University of Ghana Business School, Accra, Ghana

**Keywords:** Health systems, systems evaluation, health policy, universal health coverage, primary health care

## Abstract

**Background**: The United Nations 2030 Sustainable Development Goals have reaffirmed the international community’s commitment to maternal, newborn, and child health, with further investments in achieving quality essential service coverage and financial protection for all.

**Objective**: Using a modified version of the 1978 Tanahashi model as an analytical framework for measuring and assessing health service coverage, this paper aims to examine the system of care at the community level in Ghana’s Volta Region to highlight the continued reforms needed to achieve Universal Health Coverage.

**Methods**: The Tanahashi model evaluates health system coverage through five key measures that reflect different stages along the service provision continuum: availability of services; accessibility; initial contact with the health system; continued utilization; and quality coverage. Data from cross-sectional household and health facility surveys were used in this study. Immunization and antenatal care services were selected as tracer interventions to serve as proxies to assess systems bottlenecks.

**Results**: Financial access and quality coverage were identified as the biggest bottlenecks for both tracer indicators. Financial accessibility, measured by enrollment in Ghana’s National Health Insurance Scheme was poor with 16.94% presenting valid membership cards. Childhood immunization was high but dropped modestly from 93.8% at initial contact to 76.7% quality coverage. For antenatal care, estimates ranged from 65.9% at initial visit to 25.1% quality coverage.

**Conclusion**: Results highlight the difficulty in achieving high levels of quality service coverage and the large variations that exist within services provided at the primary care level. While vertical investments have been prioritized to benefit specific health services, a comprehensive systems approach to primary health care needs to be further strengthened to reach Ghana’s Universal Health Coverage objectives.

## Background

The United Nations 2030 Sustainable Development Goals have reaffirmed the international community’s commitment to maternal, newborn, and child health, with additional investments in achieving quality essential service coverage and financial protection for all. Universal Health Coverage (UHC), promoting the right to health for vulnerable, poor, and remote populations, has therefore become embedded as a global priority. Indeed, providing effective, affordable, and quality primary health-care services can improve maternal and child health and survival in low-and-middle-income countries (LMICs) [1–3], yet this impact is not always realized. Ongoing initiatives focusing on implementing vertical programs have constrained official efforts to evaluate overall health system coverage and strength, and understand barriers at various levels of the system [4]. Few studies provide a comprehensive overview of health systems barriers to service delivery and access to quality care at regional, district, and sub-district levels, despite the fact that service delivery in LMICs is often decentralized and focused at lower levels of care. Understanding peripheral level health systems rather than those at global or national scale is notably important in sub-Saharan Africa where regional variations within the same country can be extensive [5].

Like many countries, Ghana’s approach to UHC includes several key policy initiatives that prioritize the needs of underserved groups [6]. Ghana’s Community-based Health Planning and Services (CHPS) policy, launched and adopted in 1999, aims to reduce barriers to geographical access to health care by providing primary health care (PHC) services at the community level [7]. By posting a resident nurse in communities with an additional mandate to provide outreach services, Ghana aimed to ensure that health care reached the most vulnerable and remote populations. CHPS remains a core component of the government’s strategy and has contributed to remarkable reductions in maternal and child mortality, antenatal care (ANC) coverage, and the rising performance of immunization, particularly in underserved rural areas [8,9]. The CHPS initiative aims to leverage the strength of PHC to achieve UHC, with a notable emphasis on addressing availability and accessibility of services [10]. This expanded geographic access is enhanced by progressive policies intended to address financial barriers such as the National Health Insurance Scheme (NHIS) and user fee exemptions for services that reach pregnant women, infants, and young children [11].

### Project background

In 2016, a five-year project entitled *A National Program for Strengthening the Implementation of the Community-based Health Planning and Services (CHPS) Initiative* (CHPS+) was launched and implemented by the Ghana Health Service in collaboration with the Regional Institute for Population Studies at the University of Ghana, the University for Development Studies, and the University of Health and Allied Sciences, with technical support from Columbia University’s Mailman School of Public Health. The CHPS+ project aims to develop a sustainable capacity to implement, monitor, and evaluate health systems strength to improve national capabilities to scale-up community-based primary health-care quality and impact and achieve national UHC objectives [12]. This project aims to foster a culture of health service excellence and systems thinking previously tested by the *Ghana Essential Health Intervention Programme* in the country’s Upper East Region [10,12,13]. CHPS+ is being scaled up from the Upper East to the Northern and Volta Regions, where demonstration districts known as System Learning Districts provide models of excellence in health system delivery and implementation at the community level by strengthening the country’s community health service delivery platform.

### Objective

Using a modified version of the 1978 Tanahashi model as an analytical framework for measuring and assessing health service coverage, this paper aims to examine the system of care at the community level in CHPS+ Volta Region implementation districts to highlight the continued investments and implementation reforms needed for Ghana to achieve UHC [14].

## Methods

### Study setting

While the CHPS+ project is implemented in both the Northern and Volta regions of Ghana, this paper focuses on data from the Volta Region (VR) (now the Oti & Volta Regions). In 2010, VR had a census-enumerated population of 2.1 million, 38% of which was under the age of 15 [15]. The majority (71%) of the population is Christian. The estimated under-five mortality rate is 61 per 1000 live births, although mortality risks are known to vary by district [16]. The Volta Region is one of the poorer performing regions in key health indicators such as immunization coverage [17].

### The analytical framework: a modified Tanahashi model

The Tanahashi model, first developed in a seminal paper in 1978, was modified by UNICEF, the World Health Organization, and the World Bank in 2002 for use in the Marginal Budgeting for Bottlenecks tool to estimate the potential impact, resource needs, costs, and budgeting requirements to strengthen national health systems [14]. This adaptation, applied in analyses in more than 50 countries, is a rare framework that moves attention beyond access to health services and brings quality to the forefront, thus highlighting the effectiveness of health systems interventions and potential opportunities to optimize it [18].

This paper applies a modified version of the 1978 Tanahashi model to evaluate health system coverage at the community level through its five key measures (Table 1) that reflect different stages along the service provision continuum [19,20]. These five stages portray the complex interaction between the health system and the population in ways that can highlight gaps in service delivery [21,22]. A particularly useful element of this approach is its capability to distinguish between potential and actual coverage, highlighting gaps between available supply and demand or utilization.

**Table 1.** Tanahashi model coverage determinants

Coverage determinants	Definition
1 a. Availability of health commodities	Refers to the availability of health system inputs. These include, for example, medicines and other necessary supplies.
1 b. Availability of human resources	Refers to the availability of trained professionals at CHPS facilities that can provided needed care.
2 a. Geographic accessibility	Physical access to service delivery points.
2 b. Financial accessibility	Financial support or health insurance coverage to pay for medical care.
3. Initial contact	Refers to the first contact or use of the health services or interventions.
4. Continued utilization	Refers to the repeated contact with the health system to receive necessary care
5. Quality coverage	Represents the quality of a health intervention defined by the full course of contact with the health system to receive effective care and the minimum inputs and processes to achieve defined health effects.

*Adapted from Henriksson et al*. [22].

This analysis will utilize CHPS+ cross-sectional baseline evaluation data to assess health system strength [14,19]. Given the challenge in accessing quality data for a comprehensive picture of the primary health-care system at the community level, ‘tracer interventions’ will allow for the most relevant and local data to represent the overall system [14]. For this analysis, immunization and antenatal care data, which comprise essential components of maternal and child health and foundational elements of the CHPS initiative, will be used as tracer interventions to represent the Volta Regional Health System’s capacity to deliver essential primary health-care services [23]. We further assess sub-national service delivery by incorporating data on financial access to services in the model via individuals’ enrollment in Ghana’s National Health Insurance Scheme.

**Figure 1.**
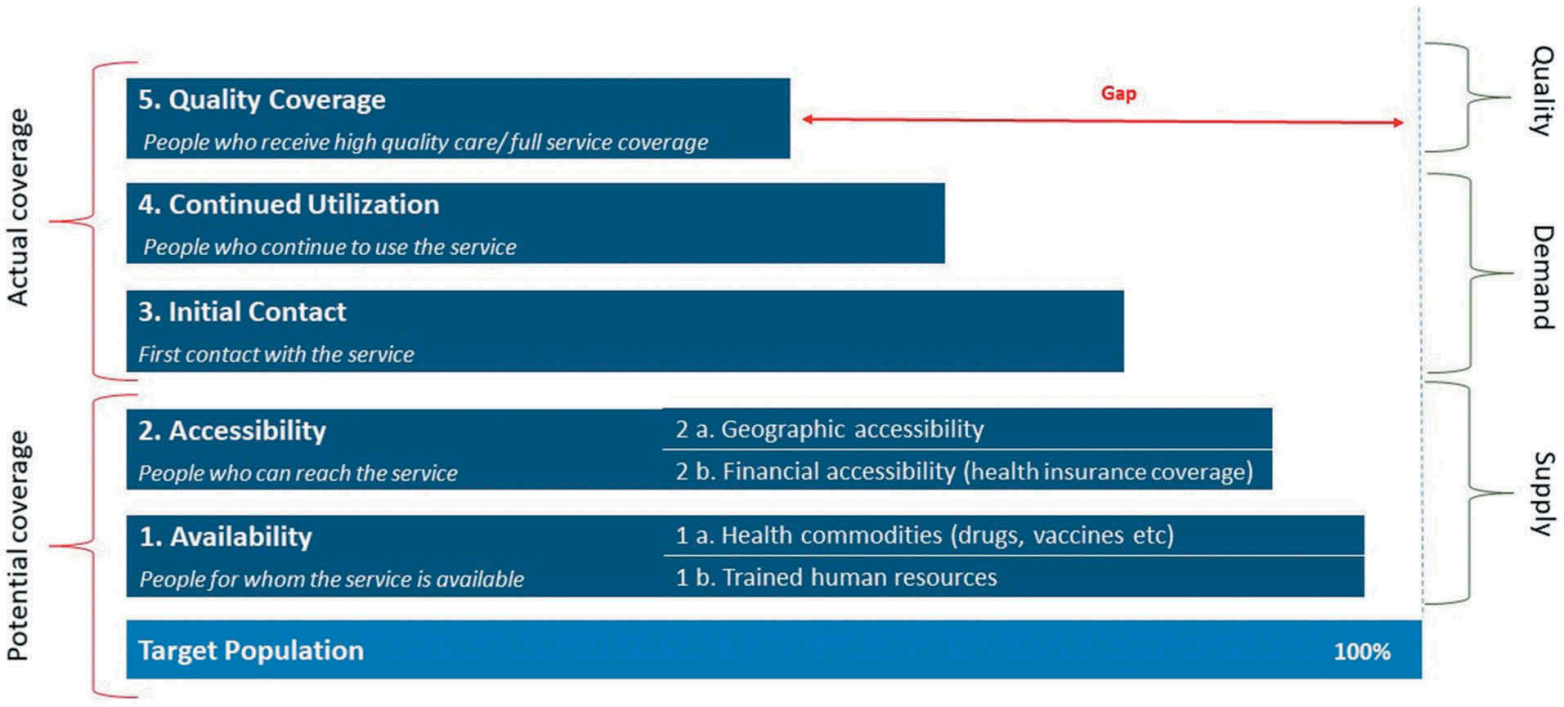
Modified Tanahashi model

The five distinct stages of the Tanahashi model (Figure 1) lend themselves to a bottom-up stepwise assessment of health services. In order to understand shortcomings in potential coverage on the supply side (availability and accessibility) and those that affect actual coverage on the demand side (initial contact, continued utilization, and quality coverage), we examine the differences between each level.

### Data sources

#### Household survey

The CHPS+ project baseline household survey was conducted in seven of the 25 districts of the VR (Central Tongu, Northern Tongu, Akatsi North, Afadzato South, Nkwanta North, Krachi East, and Krachi Nchumuru) over the April to October 2017 period. Sampling was powered to detect a 15% reduction in under-five mortality at the end of the project, with 80% power at 5% level of significance in each study region, and to draw a representative sample of women of reproductive age (15–49 years) in the CHPS+ project catchment area, collecting information on both the health of women and of their children. The survey used a two-stage stratified cluster sampling approach: the first-stage sampled enumeration areas (EAs), and the second-stage sampled households. EAs were stratified by type of location (rural or urban) and by the size of the EA (measured by the estimated number of households in the EA). EAs were also stratified by size into three groups (small, medium, or large), and subsequently sampled from each stratum using probability proportional to population size.

Immunization and antenatal care services were selected as tracer interventions to serve as proxies to assess systems bottlenecks in the study region. The dataset for both services was limited to the analysis of women and their living children aged 12 to 23 months at the time of the survey. To reduce maternal respondent recall bias, data for immunization were restricted to the interviewer’s visual assessment of each child’s immunization card.

A total of 11,201 women were interviewed in the Volta Region, 1515 of which had living children aged 12–23 months. Table 2 presents the socio-demographic characteristics of the women and their children included in the analysis.

**Table 2.** Characteristics of women and their children of 12–23 months

	Variable (mothers)	Volta region
Age	15–24	416 (27.46%)
	25–34	703 (46.40%)
	35–44	361 (23.83%)
	45–49	35 (2.31%)
Education	No Education	595 (39.27%)
	Primary	357 (23.56%)
	Middle School/JHS/JSS	473 (31.22%)
	Secondary +	90 (5.94%)
Wealth index/SES status	Poorest	245 (16.17%)
	Poorer	293 (19.34%)
	Middle	290 (19.14%)
	Richer	326 (21.52%)
	Richest	361 (23.83%)
Religion parity	Other	111 (7.33%)
	Christian	1169 (77.16%)
	Muslim	69 (4.55%)
	Traditional	166 (10.96%)
	1–3	872 (57.56%)
	4–6	461 (30.43%)
	7+	182 (12.01%)
Marital status	Single	147 (9.70%)
	Married	1076 (71.02%)
	Widowed	16 (1.06%)
	Divorced	9 (0.59%)
	Separated	70 (4.62%)
	Living together	197 (13.00%)
Occupation	No occupation	221 (14.59%)
	Student	13 (0.86%)
	Farming	795 (52.48%)
	Trading/Selling	328 (21.65%)
	Hairdressing/Dressmaking	107 (7.06%)
	Housewife	10 (0.66%)
	Other	41 (2.71%)
	**TOTAL**	1515*
	Variable (children)	Volta region
Child gender	Male	813 (52.76%)
	Female	728 (47.24%)
Place of delivery	Home	810 (52.56%)
	Govt Hospital	362 (23.49%)
	Govt Health Center	277 (17.98%)
	CHPS Compound	35 (2.27%)
	Other	57 (3.70%)
Post-natal care at the facility		686 (44.52%)
Post-natal care at home		567 (36.79%)
	**TOTAL**	1541*

*Our data set included information on 1541 children 12–23 months of age (including 28 pairs of twins) belonging to 1515 individual women.

**Table 3.** Number and facility type in the Volta Region

	7 CHPS+ Districts in VR
**Facility type**	
CHPS zone without compound	42
CHPS zone with compound	72
Health Center	31
**TOTAL**	**145**

**Table 4.** Measures for immunization and ANC as indicators of health system strength

Stage/Process component	Indicator Definition	Source
1a. Availability (commodities & equipment)	Proportion of CHPS zones and health centers (HC) with the necessary immunization services and supplies at the time of survey Proportion of CHPS facilities and HC with the necessary supplies to provide ANC, including manual BP apparatus, Sulfadoxine/pyrimethamine, iron, tetanus-toxoid vaccine, and folic acid	Health facility survey
1b. Availability (human resources)	Proportion of CHPS zones and HC with trained health personnel (CHO/CHN/EN) at the time of survey	Health facility survey
2a. Accessibility (geographic)	Proportion of the population within 5 km of a CHPS zone or HC	Household survey
2b. Accessibility (financial)	Proportion of the population with a valid National Health Insurance card	Household survey
3. Initial Contact	Proportion of children who received the BCG vaccine Proportion of women who have gone to a CHPS zone or HC for their first ANC visit	Household survey
4. Continued Utilization	Proportion of children who received all three doses of the DPT vaccine Proportion of women who have been to 4+ of their ANC visits at a CHPS zone or HC	Household survey
5. Quality/Effective coverage	Proportion of children who have received all vaccines mandated by Ghana’s Expanded Programme on Immunization (EPI) by 24 months Proportion of women who have received the minimum package of ANC services	Household Survey

#### Health facility assessment

The CHPS+ health facility assessment was conducted in July 2018 and collected information on all facilities delivering health services in the 7 CHPS+ survey districts of the VR. The survey included CHPS zones with and without functioning service posts, commonly known as ‘CHPS compounds’, Sub-district Health Centers, private clinics, and District Hospitals. For this analysis, we are limiting data to Ghana Health Service designated CHPS service catchment areas that are either equipped with or lacking functioning compounds and to government health centers (HC) (Table 3). Where services are operational, they function as the most peripheral point of provision of primary health-care services at the community level, especially in remote locations. We furthermore include the primary health-care personnel that should always be available at both CHPS zones and health centers; these include Community Health Officers (CHOs) or Community Health Nurses (CHNs), and Enrolled Nurses (EN).

The health facility survey assessed facility readiness by interviewing a health worker present at the time of interview. This provided information regarding human resources and staffing, routine care, services provided at the facility, and availability of equipment and commodities.

### Indicators

The above data sources provide an overview of the community health system in our study region. We use indicators from these CHPS+ data sources to evaluate the five stages of the modified Tanahashi model as per Table 4 below:

Data were analyzed using Stata 13; the construction of the Tanahashi model graphical representation was done in Microsoft Excel.

## Results

**Figure 2.**
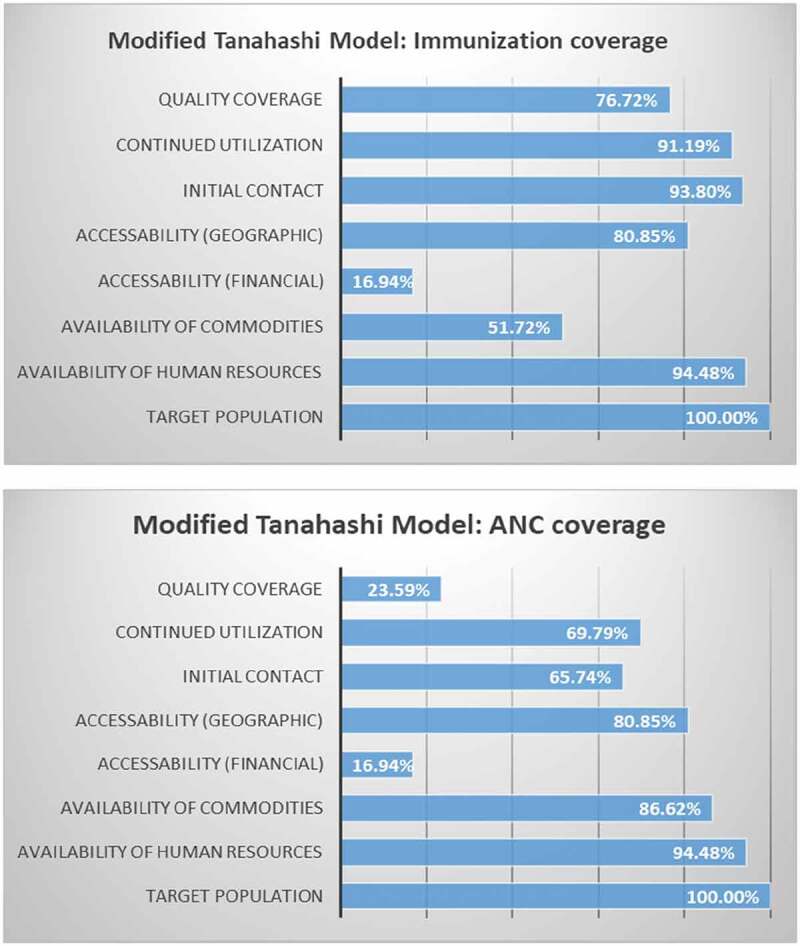
Tanahashi model results for immunization and ANC coverage

The below information provides step by step results for indicators tailored to each stage of the modified Tanahashi model for the two tracer interventions: childhood immunization and antenatal care. Figure 2 provides results according to the model.

### Tracer intervention: immunization

1a. *Availability of human resources*: The presence of a CHO/CHN at a CHPS compound or health center in our study area is 92.41%. Enrolled nurses are present in 57.93% of these community-based primary health-care centers. The overall presence of one of these health cadres is 94.48%.
1b. *Availability of essential health commodities and equipment*: Of the 145 facilities surveyed, 141 (97.24%) provide immunization services. Using vitamin A and a vaccine refrigerator as a proxy for availability of all immunization program commodities, overall availability is 51.72%.
2a. *Financial accessibility*: Of the women with children aged 12–23 months, only 16.94% had a valid NHIS card that was presented to and seen by the CHPS+ project fieldworkers.
2b.*Geographic accessibility*: Geo-located data were used to determine geographic accessibility and calculate the distance between each surveyed household and the nearest CHPS zones. Overall, 64.767% of women live within 4 km of a CHPS zone compound, as per national CHPS policy, and 29.68% of women live within 4 km of a health center. Overall, 80.85% of women live within 4 km of either a CHPS zone or a health center.
3. *Initial contact*: Overall, BCG coverage was high at 93.80%.
4. *Continued utilization*: Of the 1306 children whose immunization card was seen, 1191 (91.19%) received all three doses of a diphtheria, pertussis, and tetanus (DPT)-containing vaccine. Sensitivity analysis comparing full DPT immunization to full polio immunization as an indicator for continued utilization showed that both measures were statistically comparable with a high level of significance (p < 0.001).
5. *Quality/effective coverage*: For our immunization tracer indicator, we define effective coverage as children having received all of the basic vaccinations as required by Ghana’s Expanded Programme on Immunization (EPI), which includes one dose of BCG at birth, three doses of the oral polio vaccine (excluding the dose given at birth), three doses of a DPT-containing vaccine and hepatitis B vaccine at 6, 10, and 14 weeks, and one dose of the measles vaccine. The proportion of children with full immunization is 76.72%.

### Tracer intervention: ANC services

1a. *Availability of human resources* is same as above.
1b. *Availability of essential health commodities and equipment*: Of the 145 CHPS and health centers included in the analysis, 86.62% had the necessary commodities to provide a minimum package of ANC services, including a manual blood pressure apparatus, an adult scale, Sulfadoxine/pyrimethamine, iron tablets, tetanus-toxoid vaccine, and folic acid.
2a. *Financial accessibility* is the same as above.
2b. *Geographic accessibility* is the same as above.
3. *Initial contact*: 65.74% of women visited either a CHPS zone or HC as the location for their first ANC visit.
4. *Continued utilization*: 69.79% of women went to either a CHPS compound or a HC for at least 4 of their ANC visits.
5. *Quality/effective coverage*: For our ANC tracer indicator, effective coverage means the provision of the minimum basic package of antenatal care, including:
1. Identification of pre-existing health conditions (weight and nutrition status, anemia, hypertension)
2. Early detection of complications arising during pregnancy (pre-eclampsia, gestational diabetes)
3. Health promotion and disease prevention (tetanus toxoid vaccine, prevention and treatment of malaria, nutrition counseling, micronutrient supplements [folic acid and iron tablets], family planning counseling)
4. Birth preparedness and complication planning (birth and emergency plan, breastfeeding counseling)

The proportion of women provided with the full minimum package of ANC services at either a CHPS zone or health center is 23.59%.

## Discussion

### Assessing health system strength for UHC

The modified Tanahashi model provides an overview of the current state of the primary health-care system in seven districts of Ghana’s Volta Region. Through the application of immunization rates and antenatal visits as tracer indicators, the final models illustrate gaps in the system that remain obstacles to ensuring access, coverage, and quality health services in the VR. Results provide a deeper understanding of the provision of, and demand for, health services, as well as Ghana’s position in achieving its Universal Health Coverage ambitions.

Our data show that financial accessibility, as measured by NHIS enrollment, is poor – only 16.94% of women surveyed had valid NHIS cards. However, this did not appear to constrain the initiation of childhood immunization or antenatal care, with 93.8% and 65.7% respondents making the first visits for each service, respectively. These well-established interventions may be so highly valued by communities that women and families were willing to pay out-of-pocket [24,25] or relied on user fee exemptions to lower the potential financial access barrier. Results subsequently showed a substantial drop-off in complete quality coverage for both tracer interventions. For childhood immunizations, this was modest, dropping from 93.8% at initial contact to 76.7% quality coverage based on a composite indicator across multiple antigens. But for ANC coverage, estimates dropped from 65.7% at initial visit to 23.6% for quality coverage.

Vertical policy and financial investments in ANC and immunization have been significant over the years, and have enhanced the overall strength of the system. Ghana’s 1978 EPI operational policy aims to promote immunization delivery to all children under five through routine provision of essential vaccines at appropriate months of childhood age, and through national immunization days, and campaigns to combat specific diseases such as poliomyelitis and measles [26]. The Ghana Health Service implements this strategy through their Reaching Every District approach that utilizes the country’s decentralized health system, specifically leveraging CHPS posts and the assignment of personnel to hard-to-reach areas. Investment in immunization has likewise been very successful, with global support from international actors including GAVI – The Vaccine Alliance, committing $US 224, 538,541 between 2001 and 2016 to strengthen logistics, training, and service delivery mechanisms [27].

Moreover, investments in Ghana’s ANC program have demonstrated its importance as a component of primary health care, and a key service provided at the community level through CHPS. In 2005, Ghana’s Ministry of Health introduced free maternal healthcare delivery nationwide, of which antenatal care was a significant component. The policy aimed to reduce financial barriers to seeking maternal services [25]. In 2007, this policy was formally integrated into the NHIS, which also expanded free ANC coverage from public health facilities only to all public and accredited private and faith-based health-care providers [28]. This was found to significantly increase the percentage of children whose mothers obtained at least four ANC visits [29].

Despite policy integration to support free maternal and child health services to promote universal access to health care, the proportion of women who had verified valid insurance cards showing active membership in the NHIS was significantly lower than other reported studies [30]. Additional data from our study area show that 86% of women responded yes to having ever registered as a member of NHIS, and approximately 36% reported having valid cards that were not valid upon inspection. This likely signifies that respondents are either misinformed about their insurance status or that barriers to regularly renewing memberships – including difficulty in affording renewal payment, poor satisfaction with the services, timing of premium payment – are challenging to overcome [25]. Service delivery challenges such as inadequate registration materials may further delay renewal and exacerbate low membership [25].

Given the combined level of financial and programmatic investment in both immunization and ANC, these two health-care components should demonstrate near-perfect coverage in both the supply and demand domains of the Tanahashi model, and therefore be benchmarks for guiding the way towards achieving Universal Health Coverage. Yet, despite the fact that EPI has been integrated into CHPS, much of its funding and management have remained highly vertical. In addition, it has been possible to introduce new vaccines into the program without drastically altering the delivery method for further systems integration. These factors may contribute to the relatively higher level of quality coverage in our model. In contrast, ANC services require that peripheral health workers and subnational health systems integrate multiple funding, supply, equipment, training, and personnel streams to achieve comprehensive quality care. These complexities may account for the much larger erosion of ANC coverage after initial contact. Our findings suggest that district-level managers may be able to improve these outcomes through modest efforts that support this integration by leveraging evidence-based planning and data-driven decision-making as key guiding components of health systems strengthening.

### Strengths and limitations

Since data used for this study were collected from one of Ghana’s 10 regions, results are not generalizable to the entire country. Moreover, the Volta Region study districts were selected because they met the criteria for the CHPS+ intervention and control areas, and not because they were representative of the VR. The cross-sectional study design that is employed does not permit temporal analyses of the causes and consequences of relationships discerned for health-seeking behavior. In particular, financial access is a known determinant of care seeking that cannot be investigated.

Further, analyses are based on tools developed and data collected prior to the implementation of the CHPS+ project and the development of the current study. While estimates derived for each stage of the model were based on the best available project data, the CHPS+ household and health facility surveys were not specifically designed for the Tanahashi model. If data collection was designed for estimating the model, more specific information could have been compiled, especially regarding vaccine availability by antigen. The range of data sources required to develop this modified Tanahashi model may also not be available in settings that do not have comparable data resources.

The quality of immunization services could also have been more strictly defined as the proportion of children who have received all doses of the vaccine at the exact time and interval specified in Ghana’s EPI operational policy guidelines. However, authors remained consistent with the approach used to estimate immunization coverage in standard cross-sectional surveys such as the Demographic and Health Survey. Authors therefore looked at overall immunization completion rather than timely immunization by antigen to measure the final component of the model.

The strength of this analysis lies in the general overview it provides of the health system, guiding implementers and policymakers to look into components that require additional attention, funding, and programming to further strengthen comprehensive service delivery.

## Conclusion

The modified Tanahashi models presented above highlight the difficulty in achieving high levels of quality service coverage in the Volta Region, and the large variations that exist within services provided at the primary care level – even in the same facilities and at the hands of the same health workers and managers. In order to provide quality health-care services and reduce health disparities, policymakers must have a comprehensive grasp of the factors that constrain or enable the distribution of care, especially in reaching its most vulnerable population. This model can therefore be used as a tool to identify specific systems bottlenecks to better understand the health system, leveraging results to target financial and programmatic investments towards increasing access and achieving high quality of care. Results further strengthen the need to balance vertical global and national health investments with programming and implementation efforts focused on strengthening the overall health system in order to achieve UHC.

## Data Availability

The data that support the findings of this study are available from the corresponding author, MCS, upon reasonable request.

## Paper context

The provision of quality primary health-care services can improve maternal and child health in lower and middle income countries, yet this impact is not always realized. Previous initiatives focusing on vertical programs have constrained efforts to evaluate health system coverage and understand barriers to care. Applying the modified Tanahashi model provides a deeper understanding of the provision of, and demand for, health services, as well as Ghana’s position in achieving Universal Health Coverage ambitions.
